# Fueling of a marine-terrestrial ecosystem by a major seabird colony

**DOI:** 10.1038/s41598-020-72238-6

**Published:** 2020-09-22

**Authors:** J. Hentati-Sundberg, C. Raymond, M. Sköld, O. Svensson, B. Gustafsson, S. Bonaglia

**Affiliations:** 1 Department of Aquatic Resources, Institute of Marine Research, Swedish University of Agricultural Sciences, Turistgatan 5, 45330 Lysekil, Sweden; 2 Department of Ecology, Environment and Plant Sciences, Stockholm University, Stockholm, Sweden; 3 Baltic Nest Institute, Baltic Sea Centre, Stockholm University, Stockholm, Sweden; 4 Tvärminne Zoological Station, University of Helsinki, Hankko, Finland; 5 Department of Marine Sciences, University of Gothenburg, Gothenburg, Sweden

**Keywords:** Element cycles, Ecosystem ecology, Marine biology

## Abstract

Seabirds redistribute nutrients between different ecosystem compartments and over vast geographical areas. This nutrient transfer may impact both local ecosystems on seabird breeding islands and regional biogeochemical cycling, but these processes are seldom considered in local conservation plans or biogeochemical models. The island of Stora Karlsö in the Baltic Sea hosts the largest concentration of piscivorous seabirds in the region, and also hosts a large colony of insectivorous House martins *Delichon urbicum* adjacent to the breeding seabirds. We show that a previously reported unusually high insectivore abundance was explained by large amounts of chironomids—highly enriched in *δ*^15^N—that feed on seabird residues as larvae along rocky shores to eventually emerge as flying adults. Benthic ammonium and phosphate fluxes were up to 163% and 153% higher close to the colony (1,300 m distance) than further away (2,700 m) and the estimated nutrient release from the seabirds at were in the same order of magnitude as the loads from the largest waste-water treatment plants in the region. The trophic cascade impacting insectivorous passerines and the substantial redistribution of nutrients suggest that seabird nutrient transfer should be increasingly considered in local conservation plans and regional nutrient cycling models.

## Introduction

Animals can act as powerful biological pumps and transfer nutrients, trace elements and environmental contaminants between ecosystems^1–5^. Seabirds are arguably the most pertinent present-day vectors of compounds from marine to terrestrial ecosystems^6,7^ and have been demonstrated to enhance production and alter dynamics of local ecosystems adjacent to breeding colonies^8–10^. This general marine-terrestrial ecosystem coupling can have a number of cascade effects on biological communities. For example, seabird derived nutrients have been shown to result in complex changes on spiders and insects, and general productivity increases in terrestrial plants^10–13^. Other trophic cascades involving terrestrial-marine linkages are insectivorous passerines benefitting from salmon runs in Canadian rivers^14^, and the introduction on foxes which has diminished seabird populations and thereby changed plant communities on the Aleutian islands^15^. The diversity of pathways and often surprising effects in the above-mention studies calls for further empirical research, with a future goal of a general understanding of the role of seabirds in local ecological cascade effects and regional nutrient cycling.

The ecological significance of seabird driven nutrient fluxes have often been studied in nutrient poor terrestrial ecosystems where the addition of marine nitrogen (N) and phosphorus (P) have led to increases in productivity and diversity in plant communities^10,11,16^. The majority of the global population of seabirds breed in areas with low terrestrial productivity, and nutrient driven productivity increases have hence been interpreted to have positive effects on nutrient-limited terrestrial ecosystems^10,11^. Less focus has been given to areas where nutrients may leak to surrounding coastal ecosystems^17,18^ and especially in systems where the point sources of seabirds may aggravate already existing problems with eutrophication. Eutrophication is a widespread global problem in lakes and semi-enclosed seas^19^, and many areas affected by coastal eutrophication problems also have important seabird colonies, such as Japan and the countries around the Baltic Sea and North Sea^7^. Combatting eutrophication is an issue high on the political agenda in many areas in the world, and effective measures require a solid scientific background on bio-geochemical dynamics including the role of seabirds as nutrient vectors^19,20^.

Feather samples from juvenile piscivorous seabirds (Common murres, *Uria aalge*) and insectivorous passerines (House martins, *Delichon urbica*) collected in the largest seabird colony in the Baltic Sea, the island of Stora Karlsö have previously been shown to have a striking similarity in their *δ*^13^C isotopic ratio, suggesting a common (marine) provenance of nutrients^21^. The density of House martins on the island is also one of the highest recorded throughout the species’ distribution—and the suggested pathway for the high density has been hyperabundance of chironomids^21^. Chironomids, or non-biting midges, are insects whose larvae live in the aquatic environment and feed on microalgae, detritus and organic matter (such as seabird excrements), eventually emerging as flying adults and thus becoming available as food to insectivorous birds^22^. In this study, we clarify the ecological and bio-geochemical pathways contributing to the trophic cascade from seabirds to insectivores, by asking the following questions:

- What is the pathway by which seabird excrements feed the terrestrial foodweb?

- How does seabird excrements affect nutrient fluxes in soft bottom sediments?

- How does seabird N and P loading to the pelagic ecosystem compare to other point sources in the region?

We hypothesized that seabird nutrients would be traceble in both near-shore rocky and deep-water soft-bottom habitats, that chironomid larvae are growing up in both habitats, and that nutrient redistributed by seabirds are comparable to anthropogenic nutrient point sources in the region. The study contributes to the knowledge on ecosystem effects of seabird colonies by: (1) Exploring biological and biogeochemical pathways in which nutrient release can lead to cascade effects on terrestrial ecosystem, and (2) Describing and quantifying effects of seabird colony nutrient release in the context of an ecosystem suffering from eutrophication.

## Material and methods

### Study site

**Figure 1:**
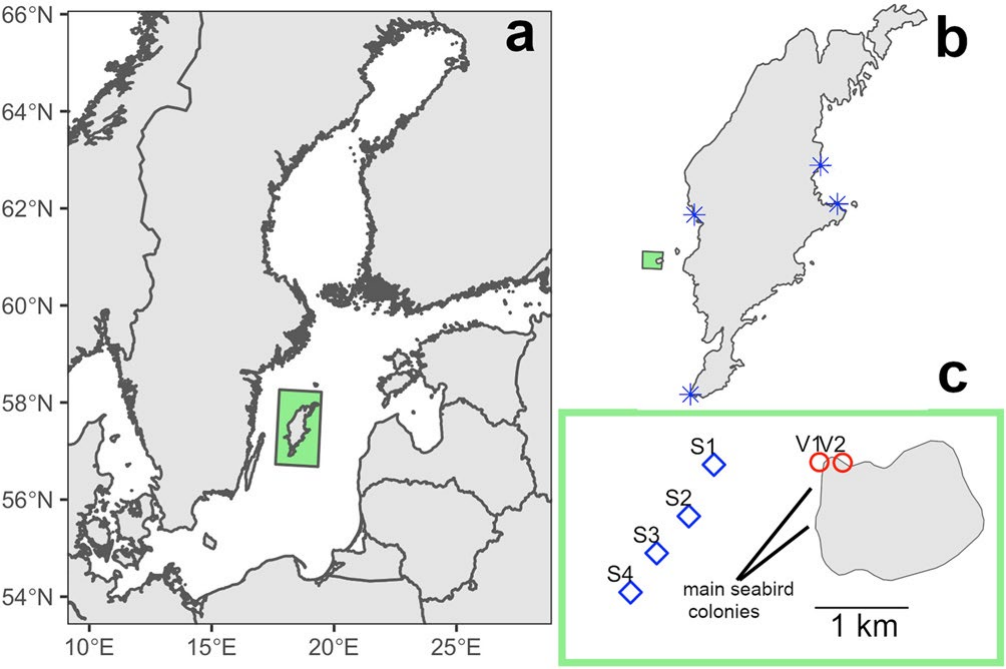
Study area. (**a**) Baltic Sea, (**b**) Island of Gotland, with Stora Karlsö indicated as a green rectangle, and sites for reference samples indicated as blue asterisks, (**c**) Island of Stora Karlsö with sediment sampling stations S1–S4 and rocky shore sampling stations V1 and V2. Guano samples were collected on the cliffs just above the V1 station.

The study was performed on the island of Stora Karlsö (57°17′N, 17°58′E) (Fig. 1), which is the largest seabird colony in the Baltic Sea^23^. The two most abundant seabird species on Stora Karlsö are Common Murre *Uria aalge* (15,700 breeding pairs) and Razorbill *Alca torda* (12,000 breeding pairs)^23^ of which approximately 9,000 pairs breed along a 300 m long narrow cliff edge indicated as V1 in Fig. 1c. The two species forage on sprat *Sprattus sprattus* and herring *Clupea harengus* and have a foraging range during the breeding period of approximately 2,000 km^2^^24^. The breeding period is from mid-April to early August and takes place on limestone cliffs 5–40 m above sea level 0–15 m from the shoreline. House martins breed on the lighthouse building on Stora Karlsö, 20 m from the cliff edge under which the seabird ledges are distributed. The number of breeding pairs in recent years have been around 150, which is one of the largest colonies in Europe of this declining species^21^.

### Water and sediment samples

Water and sediment samples were taken from onboard R/V Electra, a 24 m research vessel from Stockholm University, in April 2017. To locate suitable areas for sediment samples, two scientific echo-sounders were used, a multibeam Kongsberg EM2040, 0.4° × 0.7°, 200–400 kHz and a Kongsberg Topas PS40, 24 channels, parametric (35–45 kHz/1–10 kHz). The bottom substrate around the colony mainly consisted of hard bottom (sedimentary rocky and gravelly shores) with smaller hollows where soft sediment could accumulate. Such soft sediment areas were in the size range of 5,000 and 25,000 m^2^ and located at 1,300–2,700 m distance from the main seabird colony, and at depths of 65–69 m, and sediment sampling stations (S1–S4, Fig. 1) were located within these areas. CTD cast was performed at S1–S4 to record oxygen concentrations, temperature and salinity in the whole water column including the bottom water in situ.

### Collection and preparation of biological samples

Guano samples were taken from active breeding ledges of Common Murres in May 2017 in the Karlsö Auk lab (a man-made breeding facility for Murres and Razorbills)^25^, by scraping off material using a spatula, and then frozen to − 18 °C until preparation for isotopic analysis.

Efforts to sample macrofauna in the soft bottom sediments were performed in April 2017 on stations S1–S4. One van Veen grab (0.1 m^2^) was taken at each station and sieved through two fractions of 1 mm sieves following the European standard (ISO 16665:2014). No macrofauna was found in the samples.

Samples from the rocky shore habitat (Stations V1 and V2, Fig. 1c) were collected in April 2017 and samples at three reference sites (Fig. 1b) were collected in July and September 2018. Samples were collected by a snorkeler that scraped macroalgae into a bag (mesh size < 0.5 mm) taking 6–14 subsamples from each station and preserving the contents in 90% ethanol. The major macroalgae taxa were *Fucus vesiculosus* and *Cladophora glomerata*. Chironomid larvae was sorted out from the macroalgae subsamples in the laboratory for isotopic analyses, where the isotopic signal of each subsample was analysed separately to obtain averages by station. Stations V1 and V2 were at a distance of 10 and 500 m from the main seabird colony, respectively, whereas the distance to the reference stations all exceeded 15,000 m. All samples were collected at a distance from shore of 2 – 10 m. The Baltic Sea has no tide.

### Sediment coring procedures

Sediments at S1–S4 were sampled by means of a Kajak corer (tube length: 50 cm, internal diameter: 8 cm), which provided nearly undisturbed sediment surface. At each station, one bottom water sample was taken from the Kajak core and the sediment was then immediately sliced at the following resolution: 1 cm slices for the first 4 cm, and then 2 cm slices from 4 to 10 cm. Each sediment slice was transferred into a 50 mL Falcon tube and centrifuged at 670×*g* (2,500 rpm) for 15 min to extract porewater. The bottom water and porewater samples were collected with a clean plastic syringe and filtered (0.45 μm polyethersulfone filter) into a 10 mL polypropylene tube. The tubes were stored at − 20 °C until later analyses of dissolved ammonium (NH_4_^+^), phosphate (PO_4_^3−^) and nitrate plus nitrite (NO_3_^−^ + NO_2_^−^). At each station, a second Kajak core was sliced with resolution of 1 cm slices for the first 4 cm and samples were stored at -20 °C for later determination of organic geochemistry parameters (org C, N, *δ*^13^C, *δ*^15^N signatures and sediment porosity).

### Isotopic ratio (*δ*^13^C and *δ*^15^N) analyses of biological and sediment samples

Sediment and biological (bird faeces and chironomid larvae) samples were freeze dried, ground, homogenized and weighed in tin capsules. Analyses on biological samples were performed with a PDZ Europa ANCA-GSL elemental analyzer (1,000 °C combustion) connected to a PDZ Europa 20-20 isotope ratio mass spectrometer (IRMS, Sercon Ltd.), while sediment samples were analyzed on an Elementar Vario EL Cube elemental analyzer (Elementar Analysensysteme GmbH) (1,080 °C combustion) connected to the same IRMS system. Isotopic compositions were reported using the conventional *δ* notation^26^, which reports the isotopic composition of a sample as the ‰ deviation of a sample relative to Vienna Peedee belemnite (VPDB) for *δ*^13^C and to atmospheric N_2_ for *δ*^15^N. Samples were regularly interspersed with two different laboratory standards, which were previously calibrated against NIST Standard Reference Materials (IAEA-600, USGS-40, USGS-41, USGS-42, USGS-43, USGS-61, USGS-64 and USGS-65). Based on the analyses of these standards the analytical precision was ± 0.2 ‰ for *δ*^13^C and ± 0.3 ‰ for *δ*^15^N.

### Porewater analyses and diffusive flux calculations

Porewater samples were thawed, diluted 1:10 and NH_4_^+^, NO_x_ (= NO_3_^−^ + NO_2_^−^) and PO_4_^3−^ were analyzed on a segmented flow autoanalyzer system (ALPKEM, Flow Solution IV) following the standard methods for seawater analyses^27^. Precision was ± 0.036 µmol L^−1^ for NH_4_^+^,  ± 0.014 µmol L^−1^ for NO_x_ and ± 0.016 µmol L^−1^ for PO_4_^3−^. As there was no macrofauna in the sediment, porewater profile shapes could be easily modeled to calculate diffusive fluxes of NH_4_^+^ and PO_4_^3−^, while NO_x_ profiles did not show any generalizable trend and were not modeled. Profiles were modeled with the numerical interpretation by Berg et al.^28^, which provides the best fit to a measured concentration profile assuming steady state conditions and returns diffusive fluxes between the sediment–water interface (SWI) as a function of depth. We assumed that biological diffusivity (movement of solutes due to bioturbation) was zero as macrofauna was absent, so that diffusive sediment–water fluxes (*J*) could be calculated according to Fick’s First Law of diffusion:

1 $$J=-\varphi Ds\frac{\delta C}{\delta x}$$

where *φ* is sediment porosity, *Ds* is molecular diffusivity in sediment, *C* is the solute concentration determined analytically and *x* is sediment depth. Porosity was estimated from sediment water content, which was calculated by measuring the wet and dry weight of 5 mL sediment aliquots after drying them at 105 °C. *Ds* was calculated according to the equations reported by Iversen and Jørgensen^29^.

### Quantification of total nutrient emissions

We compiled literature values on guano production as well as nitrogen and phosphorus concentration in guano to calculate the nitrogen and phosphorus emissions from the colony:

2 $${\text{Em}}_{i} = {\text{Pop}}*{\text{DG}}*{\text{PC}}*{\text{Conc}}_{i}$$

where Em is the total emissions of nutrient *i* (nitrogen and phosphorus) [g], Pop is the population size of the two seabird species, DG is the Daily guano output per individual and day [g day^−1^ dry mass], PC is the time each seabird individual spent in the colony, and Conc is the concentration of nutrient *i* in bird faeces. Population sizes were taken from a recent census^23^ and time spent in the colony was estimated from observation studies of adult birds feeding chicks^30^. Daily guano production was taken from previously reported data on Thick-billed murre, a closely related species of the same breeding ecology and size as Common murre^31^. Nutrient content in guano was taken from a number of published studies on seabirds (Table S1) where we used the median, 5th and 95th percentiles to calculated confidence intervals for the daily seabird nutrient emissions.

## Results

### Seabird impacts on chironomid abundance

**Figure 2:**
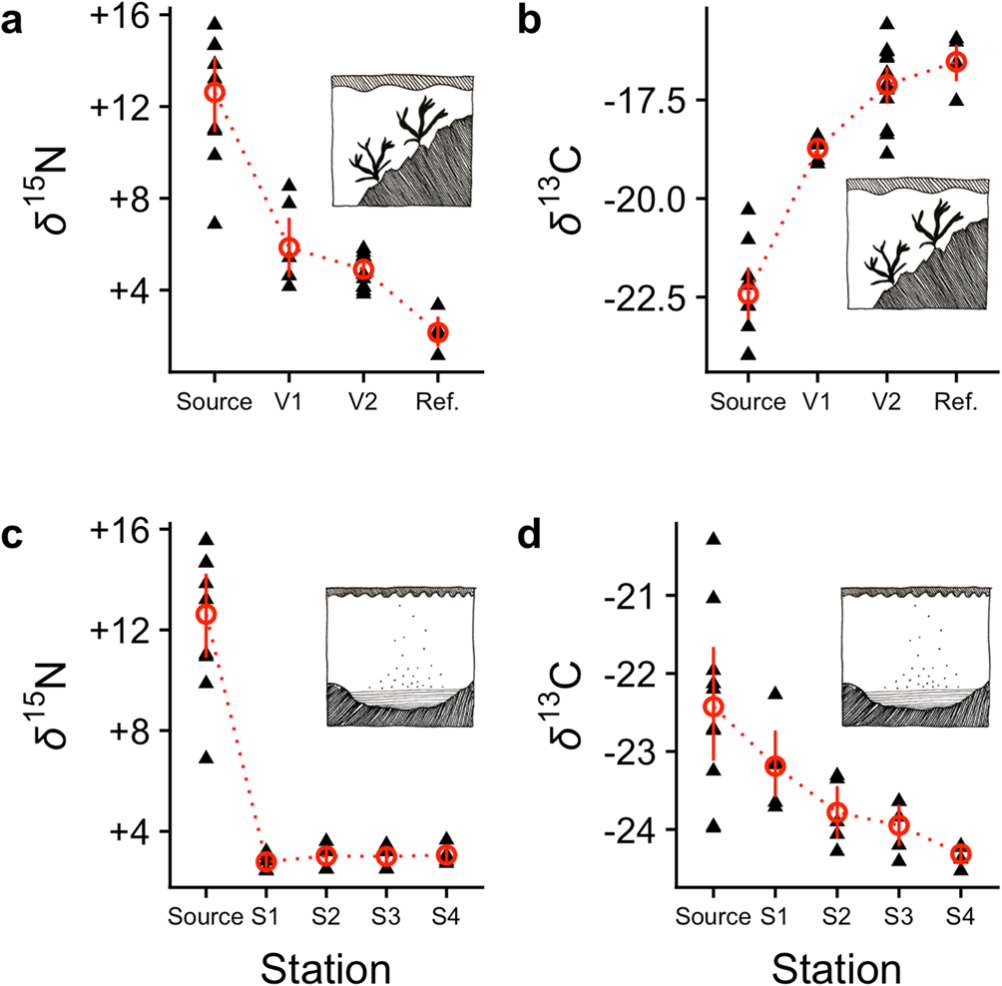
Stable isotopes as a function of distance to the seabird colony. (**a**) *δ*^15^N in chironomids (rocky shore habitat), (**b**) *δ*^13^C in chironomids (rocky shore habitat), (**c**) *δ*^15^N in sediments, and (**d**) *δ*^13^C in sediments. “Source” refers to seabird guano sampled inside the colony. “Ref.” refers to reference samples (island of Gotland, Fig. 1b). Black triangles indicate raw data at different stations and red circles denote mean by station. For station description see Fig. 1c. All *δ* values are given as ‰.

The samples of macroalgae near the seabird colony included large numbers of Chironomidae larvae which were highly enriched in *δ*^15^N (Fig. 2a). Also the *δ*^13^C signal was seen as a gradient with increasing values at increasing distance from the colony (Fig. 2b). The deep-water soft bottom sediments did not host any living macrofauna at all. We observed no effect of distance to the bird colony on the *δ*^15^N signal in the sediments (Fig. 2c) whereas the *δ*^13^C signal was seen as a gradient with decreasing values at incresing distance from the colony (Fig. 2d). Although the oxygen concentrations measured would not prevent the presence of typical Baltic Sea macrofauna in the sediments, monthly data on dissolved oxygen from a nearby oceanographic sampling station (57.116 N, 17.667 E) revealed anoxic events in early 2016 and possibly also in mid 2016, i.e. 1–1.5 year prior to the benthic sampling in this study (Fig. S1).

### Porewater nutrient profiles and fluxes in soft-bottom sediments

**Figure 3:**
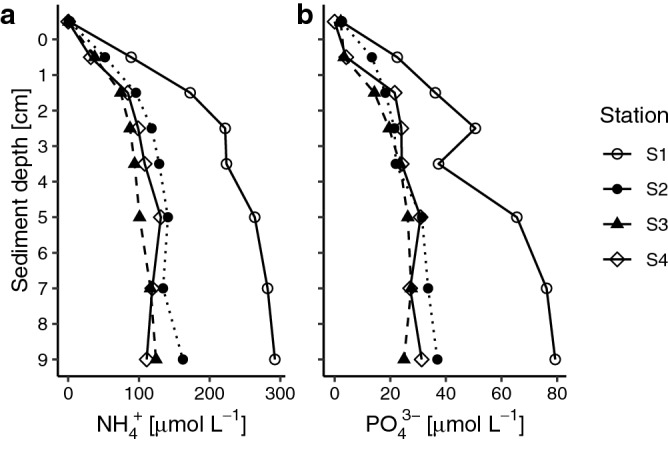
Nutrient concentrations in sediments at different sediment depths. (**a**) NH_4_^+^ and (**b**) PO_4_^3−^. − 0.5 cm (top data value) refers to bottom water.

Porewater NH_4_^+^ and PO_4_^3−^ concentrations increased with depth in the sediment and were highly elevated at the sediment station closest to the seabird colony (S1) reaching ca. 300 and 80 µmol L^−1^ at 9 cm depth, respectively (Fig. 3). Concentrations of the two solutes were on a lower level at the three other stations (S2‒S4), where they did not exceed 170 and 40 µmol L^−1^ at 9 cm depth, respectively (Fig. 3). Porewater NO_x_^−^ concentrations were negligible and even in the top oxidized layer (0.5 cm layer) they were < 1.5 µmol L^−1^ (data not shown). Bottom waters at all sediment stations were low in oxygen (1.7–2.4 ml L^−1^) but not anoxic. The four stations had similar salinity (8.1–8.2‰) and water temperature (5.0–5.1 °C).

**Figure 4:**
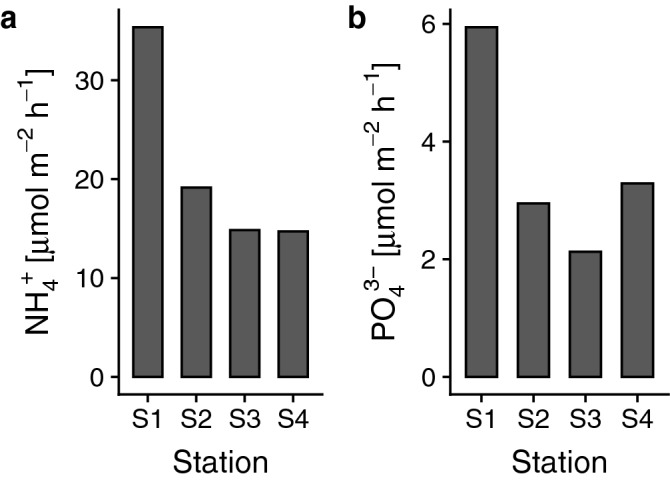
Modelled fluxes in soft-bottom sediments. (**a**) NH_4_^+^, and (**b**) PO_4_^3−^.

The diffusive fluxes of both NH_4_^+^ and PO_4_^3−^ were higher at S1 compared to the other three stations (S2‒S4) (Fig. 4), suggesting a stronger release of nutrients in proximity of the colony compared to the more offshore stations.

### Nutrient release from seabirds

**Figure 5:**
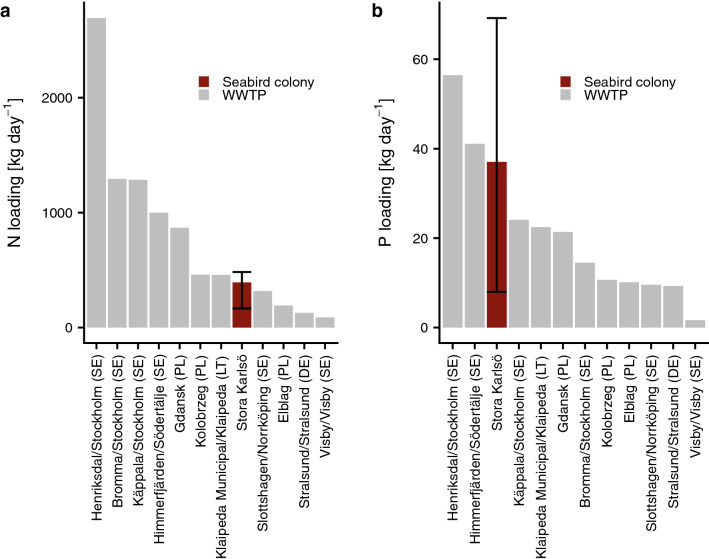
Estimated daily nutrient release from the Stora Karlsö seabird colony compared to other major Baltic Sea point sources (Waste-water treatment plants, WWTP). (**a**) nitrogen, and (**b**) phosphorus. WWTP data were obtained from^32^. Abbreviations in x-labels: SE = Sweden, PL = Poland, LT = Lithuania, DE = Germany.

Daily release from seabirds in the colony were estimated at 393 kg N day^−1^, 95% C.I. [166–483], and 37 kg P day^−1^ 95% C.I. [8–69] (Fig. 5). The wide confidence intervals are caused by the large variation in the estimations of N and P concentrations of seabird guano (Table S1). Nevertheless, the numbers indicate that the order of magnitude of the seabirds’ P emissions are similar to those of the largest waste water treatment plants and N emissions are in the same magnitude as a number of mid size treatment plants in the Baltic Sea (Fig. 5).

## Discussion

**Figure 6:**
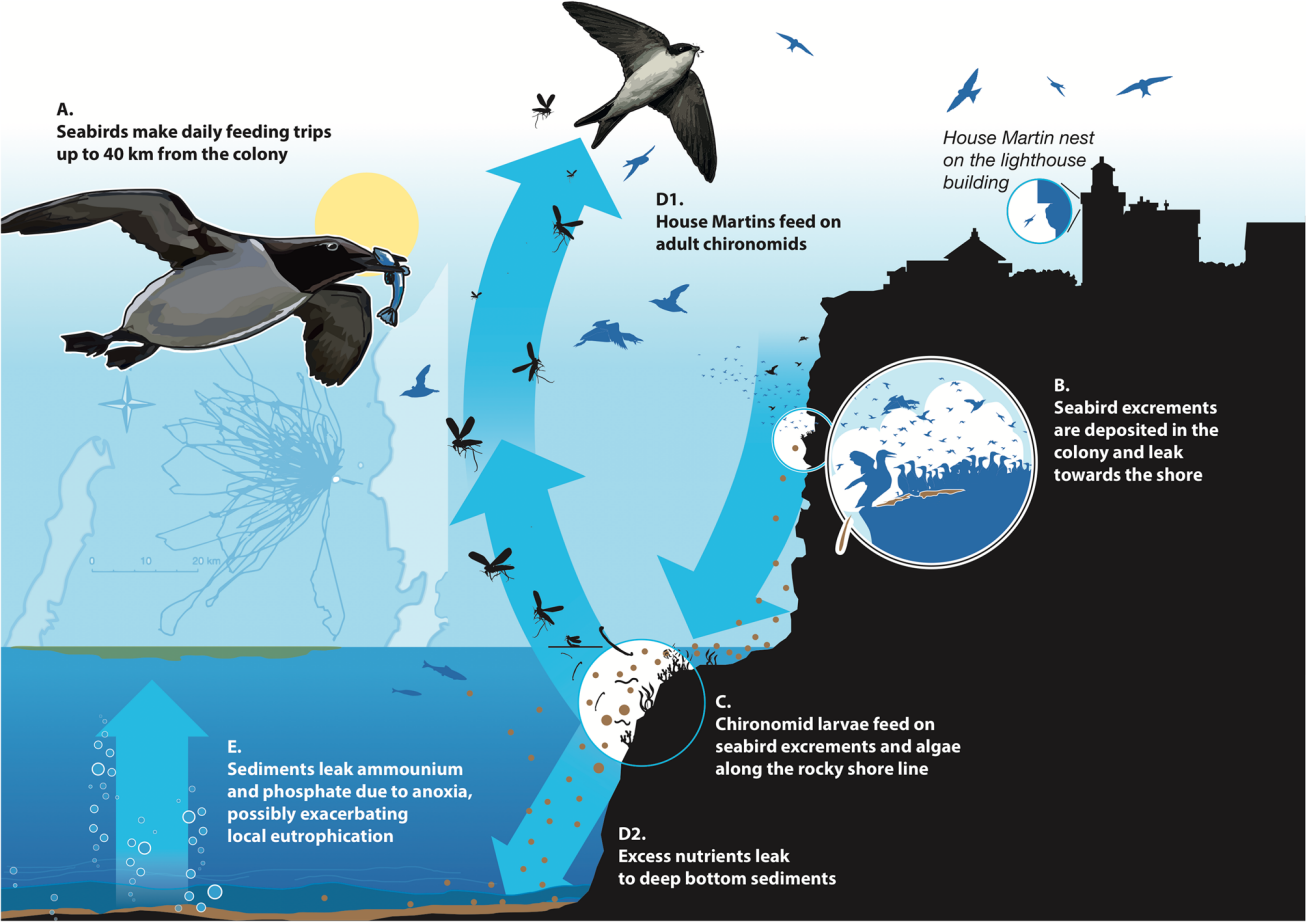
Conceptual visualization of the main processes investigated in this paper. The Stora Karlsö lighthouse with house martin nests are shown at the top right, with the seabird colony located at the cliff edges at the shoreside. Illustration by Fredrik Saarkoppel / Kobolt Media AB.

Seabirds can act as powerful biological pumps by moving chemical compounds across wide distances and are thereby significant drivers of biogeochemical cycling^7^ and biovectors of environmental contaminants^6^. We show that N and P release from a seabird colony in the eutrophic Baltic Sea is in the same order of magnitude as waste water treatment plants^32^, and thereby a driver of regional nutrient cycling. These significant releases have constrasting effects in two habitats. Along rocky shores, the release lead to high production of chironomid larvae, and thereby enhanced food supply to insectivorous House martins breeding on the island that feed on adult (flying) chironomids^21^. In deep-water sediments surrounding the colony, nutrient release did not support macrofaunal production, probably because of seasonal anoxia, and these sediments acted as sources of dissolved N and P to the water column. These contrasting effects on different ecosystem compartments arise from dynamics at different spatial and temporal scales that interact in a complex manner (Fig. 6).

### How do seabirds support increased insect production?

This study was originally conceived based on an observation of the unusually large colony of House martins (i.e., insectivorous passerines) on the lighthouse adjacent to the seabird colony (Fig. 6), something that later was linked to the seabirds via isotopic analysis of feather samples^21^. Here we expand on these findings by investigating the pathways by which the seabird derived nutrients affect the surrounding terrestrial and marine ecosystems. We found that the nutrients can be traced both along rocky shores and in deep-water sediments, but it was only along the rocky shores that the nutrients supported chironomid production. The isotopic signature of N (*δ*^15^N) in chironomids in the rocky shores show a decreasing trend with distance from the colony, which indicates seabird derived nutrients in the chironomid production. Our visual observations from the island suggest extremely high concentrations of flying chironomids during spring months, which is probably supporting not only House martins but insectivore bird species on the island in general. The seabirds are thus increasing the availability of nutrients in support of local biological production in the near-shore marine habitats, which cascades back to the terrestrial (island) ecosystem. Our results reinforce earlier studies that have shown strong and sometimes unexpected effects of seabirds in local flora and fauna^10–13^. We believe that such seabird mediated cross-scale ecosystem interactions are often overlooked, and should be considered more generally, e.g., in constructing management plans of protected areas^33–35^.

### Seabird effects on sediment fluxes

The deep-water sediments near the seabird colony were strongly enriched in nutrients but did not support any macrofaunal production. Although our measured oxygen concentrations would not prevent the presence of typical Baltic Sea macrofauna, regularly returning anoxic events preceeding the sampling was the probable reason behind the lack of macrofauna in this habitat. Since we sieved the sediments with 1.0 mm sieves, we cannot exclude that smaller invertebrates (e.g., meiofauna, larval stages of macrofaunal organisms, etc.) were actually living in these sediments, but it is not the focus of this study as they cannot explain the link between the marine habitat and terrestrial birds. Another potential explanation for the lack of chironomids in the sediments would be that larval growth occurs later in the season, however, chironomids are known to have extended growing seasons with overlapping generation and are thus expected to be detected throughout the year^36^, and the emergence of adults in the Baltic Sea occurs in May and June^37^ which means that larvae, if present in the habitat, would be detected in April.

The nutrient data alone cannot say whether there was a direct fertilization effect by the birds or if the enrichment was due to some indirect effects. However, the δ^13^C signals in the guano were very similar to those in the sediment samples close to the station, which strongly indicate a direct C fertilization effect. The observed gradient with decreasing δ^13^C at increasing distance from the colony could theoretically be an effect of decreasing input of terrestrial dissolved organic matter (DOM) ^38,39^. However, the type of benthic ecosystem at our offshore study site is thought to be much more affected by benthic-pelagic coupling than by terrestrial DOM input^40^ by which we can be relatively certain that the δ^13^C is a function of colony distance rather than general DOM input variability.

The *δ*^15^N signals in the sediments (ca. + 3 ‰) were lower than those from Baltic coastal settings affected by human sewage discharge (+ 7‒8‰) as reported by Bonaglia et al.^41^. The low values of the present study are likely supported by high contribution of surface N_2_-fixing cyanobacterial blooms and subsequent deposition of this biomass. There is a strong link between hypoxia and cyanobacterial blooms in the Baltic Sea^42^. Altogether, this suggests that N fertilization from bird guano was not reflected in N burial, but was rather supporting N recycling, which exacerbates hypoxia and cyanobacteria blooms.

Our nutrient data indicate that the near-colony sediments were acting as stronger sources of both nitrogen and phosphorus to the water mass than sediments further away. This was due to the combination of high ammonium and phosphate concentrations in the porewater environment, and low oxygen. With more oxygen, the essential nutrients such as phosphate would remain in the sediments and possibly lead to biological production including chironomid larvae, i.e. a strengthened link between the seabirds and the surrounding benthic and terrestrial ecosystem compartments^43^. With even less oxygen than under present conditions, there would be more phosphate and ammonium present both in the sediment porewater and in the water column as these compounds would be prevented from precipitating (PO_4_^3−^), oxidizing (NH_4_^+^) or binding to sediment particles (NH_4_^+^), and they would tend to leave even more from the sediment to the water phase than under present conditions^44^. In Baltic Sea sediments affected by hypoxia, NH_4_^+^ is generally produced at high rates in the sediment and efficiently exchanged to the water column because of high rates of dissimilatory reduction to ammonium (DNRA), anaerobic mineralization of organic matter and ammonification^41^. In these conditions, additionally, NH_4_^+^ adsorption to sediment particles is generally limited since water content is extremely high and the sites of NH_4_^+^ exchange likely saturated^45^ . In hypoxic Baltic sediments, phosphate desorption from iron oxyhydroxides leads to high sediment–water fluxes of PO_4_^3−^ and generally high PO_4_^3−^ concentrations in the bottom water^42^.

### Seabird fertilization in relation to regional nutrient cycling

The majority of studies on seabird colony nutrient enrichment has been performed in nutrient poor ecosystems^10,11,16^. We study a strongly eutrophic system, an offshore area in the Baltic Sea, where under normal circumstances a high proportion of the biological production in the pelagic ecosystem sinks to the bottom^40^, and contributes to oxygen consumption at depths of 60‒70 m and below^46^. Seabirds’ foraging movements thus release the effect of eutrophication from their foraging areas by removing pelagic fish biomass and at the same time contributing to eutrophication around the colony (Fig. 6). The estimated daily release from the seabird colony of 393 kg N and 37 kg P day^−1^ assumes that all the N and P excreted as guano by the birds ends up in the Baltic Sea. However, a part of these nutrients, especially the very reactive N, will be lost via volatilization of ammonia and especially by denitrification in the anoxic, deposited faeces on the island^7^. In case of significant denitrification happening in the guano, the N content should be < 10% according to a study from cave guano^47^. However, the N% of our guano samples and especially the average value (11.2%) was above that threshold. We thus conclude that overall decomposition and N loss were negligible if compared to the quantity of N leaking into the Baltic Sea.

The total emission from the colony is in the same order of magnitude as the major anthropogenic point sources in the Baltic Sea, keeping in mind that the birds do not add nutrients to the system but concentrate them to small and distinct areas. Nevertheless, internal nutrient fluxes in the Baltic Sea are of similar magnitude or even larger than external inputs^48^. Thus, our findings suggest the need for considering previously overlooked dynamics between nutrient cycling such as seabird foraging especially when studying processes on smaller (< 10,000 km^2^) spatial scales.

## Conclusions

Colonial seabirds forage over vast geographic areas but release the majority of their excrements at their colonies, leading in our case to local nutrient release in the order of magnitude as major point sources considered in management plans to mitigate eutrophication. We can track the effect of this nutrient release in two completely different habitats adjacent to the colony (macroalgae growing along rocky shores and muddy deep-water sediments), where they lead to contrasting effects on biological production. The magnitude of the seabird nutrient transfer and local enrichment motivates increased consideration of these processes in regional biogeochemical modelling. Furthermore, our results suggest that the success of terrestrial biodiversity conservation on seabird islands may be conditioned by the supply of marine derived nutrients, which calls for a better integration between marine and terrestrial management and conservation plans. Modelling studies suggest that seabird’s nutrients transfers are globally significant, but the ramifications of these cross-scale ecosystem linkages for terrestrial ecosystem management and conservation are yet to be described and quantified.

## Supplementary information

Supplementary file1

## Data Availability

The datasets generated from this study are available in the Mendeley data repository, https://doi.org/10.17632/vzt7cj9th5.2 and https://doi.org/10.17632/dj9pnpdv8d.1
