# Supplementary material for: Fueling of a marine-terrestrial ecosystem by a major seabird colony
Source: Sci Rep. 2020 Sep 22;10:15455. doi: 10.1038/s41598-020-72238-6 (PMC7508978; doi:10.1038/s41598-020-72238-6)
Supplement: Supplementary file 1 — Supplementary file1 [file 41598_2020_72238_MOESM1_ESM.docx]

**Fueling of a marine-terrestrial ecosystem by a major seabird colony**

Hentati-Sundberg, J., Raymond C. , Sköld, M., Svensson, O., Gustafsson, B. & Bonaglia, S.

**SUPPORTING INFORMATION**

**Calculation of seabird nutrient emissions**

**Table S1.** N and P concentrations in seabird feaces from a number of previous studies.

| **Species** | **Dataset** | **% N** | **% P** | **Reference** |
| --- | --- | --- | --- | --- |
| Common guillemot |  | 22.2 |  | ^1^ |
| Black guillemot |  | 21.6 |  | ^1^ |
| King penguin |  | 20.00 | 0.4 | ^2^ |
| Macaroni penguin 1 |  | 21.54 | 0.362 | ^2^ |
| Macaroni penguin 2 |  | 21.64 | 0.358 | ^2^ |
| *Commercial guano* |  | 11.70 | 2.6 | ^3^ |
| *Tropical seabirds* |  | 18.25 | 3.75 | ^4^ |
| *Temperate seabirds* |  | 9.00 |  | ^5^ |
| *Temperate seabirds* |  | 18.00 |  | ^5^ |
| Westland petrel | June | 3.00 | 1.62 | ^6^ |
| Westland petrel | November | 12.80 | 1.72 | ^6^ |
| Blue petrel |  | 15.83 | 2.08 | ^7^ |
| Great-winged petrel |  | 16.20 | 1.14 | ^7^ |
| Soft plumaged petrel |  | 17.39 | 1.79 | ^7^ |
| Grey petrel |  | 19.66 | 1.36 | ^7^ |
| White-chinned petrel |  | 14.72 | 2.57 | ^7^ |
|  |  |  |  |  |
| **Median** |  | **17.70** | **1.67** |  |
| **5 percentile** |  | **7.35** | **0.364** |  |
| **95 percentile** |  | **21.78** | **3.12** |  |

**Time series of oxygen concentrations**

**
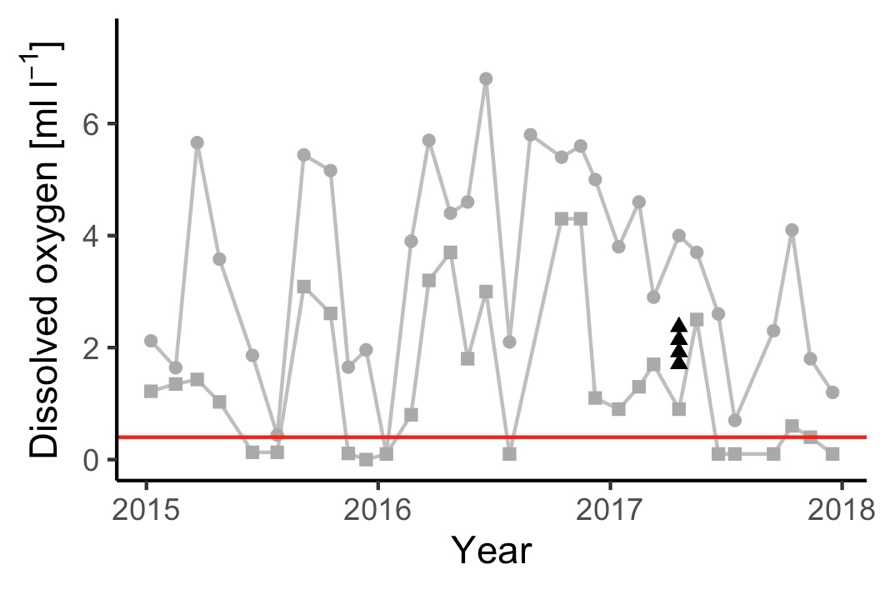
**

**Fig. S1.** Timeseries of dissolved oxygen near the seabird colony. Grey points are data for the oceanographic sampling station “BY 38 Karlso” (57.116 N, 17.667 E) at 60 m depth (circles) and 70 m depth (squares), and black triangles are at 65 - 69 m depth at the four stations reported in this study. The red horizontal line indicates 0.4 ml/l oxygen i.e. the level below which no macrofauna will survive^8^.

**References**

1. Brekke, B. & Gabrielsen, G. W. Assimilation efficiency of adult Kittiwakes and Brünnich’s Guillemots fed Capelin and Arctic Cod. *Polar Biol.* **14**, 279–284 (1994).

2. Lindeboom, H. J. The Nitrogen Pathway in a Penguin Rookery. *Ecology* **65**, 269–277 (1984).

3. Hartz, T. K. & Johnstone, P. R. Nitrogen availability from high-nitrogen-containing organic fertilizers. *Horttechnology* **16**, 39–42 (2006).

4. Smith, J. S. & Johnson, C. R. Nutrient inputs from seabirds and humans on a populated coral cay. *Mar. Ecol. Prog. Ser.* **124**, 189–200 (1995).

5. Gillham, M. E. Ecology of the Pembrokeshire Islands: V. Manuring by the Colonial Seabirds and Mammals, with a Note on Seed Distribution by Gulls. *J. Ecol.* **44**, 429 (1956).

6. Hawke, D. J. Soil P in a forested seabird colony: Inventories, parent material contributions, and N:P stoichiometry. *Aust. J. Soil Res.* **43**, 957–962 (2005).

7. Fugler, S. R. Chemical Composition of Guano of Burrowing Petrel Chicks (Procellariidae) at Marion Island. in *Siegfried W.R., Condy P.R., Laws R.M. (eds) Antarctic Nutrient Cycles and Food Webs. Springer, Berlin, Heidelberg* 169–179 (1985).

8. Diaz, R. J. & Rosenberg, R. Marine benthic hypoxia: a review of its ecological effects and the behavioural responses of benthic macrofauna. *Oceanogr. Mar. Biol. an Annu. Rev. Vol. 33* 245–303 (1995).
